# Editorial

**DOI:** 10.1107/S1600536809051757

**Published:** 2009-12-19

**Authors:** William T. A. Harrison, Jim Simpson, Matthias Weil

**Affiliations:** a Department of Chemistry, University of Aberdeen, Aberdeen AB24 3UE, Scotland; b Department of Chemistry, University of Otago, PO Box 56, Dunedin, New Zealand; c Institute of Chemical Technologies and Analytics, Division of Structural Chemistry, Vienna University of Technology, Getreidemarkt 9/164-SC, Austria

Regrettably, this editorial is to alert readers and authors of *Acta Crystallographica Section E* and the wider scientific community to the fact that we have recently uncovered evidence for an extensive series of scientific frauds involving papers published in the journal, principally during 2007. Although several thousands of structures published in *Acta Crystallographica Section E* every year will continue to reflect results of serious scientific work, the extent of these problems is significant with at least 70 structures demonstrated to be falsified and meanwhile acknowledged by the authors as such. Our work is ongoing and it is likely that this figure will rise further.

These problems were first discovered by Ton Spek during testing of the checking programs for the journal. Testing is routinely carried out using cifs and structure-factor files from back issues of *Acta Crystallographica Sections E* or *C*. Initially, unexplained Hirshfeld rigid-bond alerts and unusual metal–ligand donor-atom distances led to the discovery that metal atoms had been transposed and that more than one structure had been ‘determined’ using identical sets of data. Investigation of these cases sparked a search of papers written by the correspondence authors involved.

A program written by Toine Schreurs of Utrecht University that can examine and compare two structure-factor files was then used to examine the data deposited for the structures under investigation. For all of the problem structures, the program revealed that the data sets used to refine two or more supposedly unique structures were in fact identical, but with the cell parameters apparently manually altered by the authors concerned.

The falsified structures have many features in common: in each case, a *bona fide* set of intensity data, usually on a compound whose structure had been correctly determined and reported in the literature, was used to produce a number of papers, with the authors changing one or more atoms in the structure to produce what appeared to be a genuine structure determination of a new compound. The worst example generated no fewer than 18 supposedly original structures from a single common set of data. There is nothing to suggest that the authors of the original papers describing the real structures are in any way aware of, or complicit in, this fraud.

Bogus refinements were found for both metal-organic and organic structures. The most common ploy was to acquire a data set for a coordination complex, say of copper(II). Changing the metal from copper(II) to zinc(II), nickel(II), iron(II) or even cobalt(III) produced papers reporting seemingly novel compounds. In order to decrease the risk of detection, changes in the metal were generally accompanied by small (< 4%) manual alterations to the unit-cell parameters and also the culling of some reflections from the data sets. The scale of the problems ruled out the possibility of mere incompetence.

Similar procedures with structures containing lanthanide elements offered even greater scope for deception. In addition to changing the identity of the metal, alterations to atoms in the organic ligands added further variation to the structures falsely reported.

Non-metal atom substitutions also generated numerous bogus organic structures. CH_2_ groups were replaced by NH or O and *vice versa*, nitro groups became carboxylic acids and amides, OH groups became fluorine atoms; the list is extensive. The residuals on the resulting fraudulent refinements were generally worse than those of the genuine material but not sufficiently so as to cause undue concern on their own. However, chemically implausible or impossible structures arose from these manipulations, and it is a concern and disappointment that these chemical features passed into the literature undetected.

The initial set of falsified structures arises from two groups. The correspondence authors are Dr H. Zhong and Professor T. Liu, both from Jinggangshan University, Jian, China. The co-authors on these papers included other workers from Jinggangshan University together with authors from different institutions in China. Both these correspondence authors and all co-authors have signed forms agreeing to the retraction of 41 papers published by Dr Zhong and 29 by Professor Liu. Details of these retractions appear elsewhere in this issue of the journal. Having found these problems with articles from Jinggangshan University, all submissions from this University to *Acta Crystallographica* Sections *E* or *C* have now been identified and are being checked for authenticity. Preliminary results indicate that further retractions will result from this exercise.

All Co-editors of *Acta Crystallographica Sections* *E* and *C* have been alerted to these fraudulent practices and have been advised of the warning signs that can be used in most instances to identify such attempts to deceive. It should be noted that many other possibly fraudulent submissions were rejected at the refereeing stage by alert and conscientious Co-editors, but until the scope of the fraud became apparent, these were reasonably regarded as one-off examples of incompetence or honest mistakes.

When we discussed the events with the Editors of other journals in the *Acta* family, they expressed amazement, because, like us, they assumed that it was almost inconceivable that a fake crystal structure would be submitted for publication. Sadly, that has proven not to be the case and we must now take stock and decide what steps are needed to prevent further scientific fraud. To that end, the *checkCIF* validation software is being improved continuously and provides an exhaustive assessment of data and structural quality and consistency. It is also noteworthy to point out that the current problems could not have been easily discovered without the availability of the structure-factor files; it will become increasingly important for all journals reporting crystal structures to make sure that they require authors to supply such data in future.

Finally, nothing can replace the sceptical (but fair) assessment of an experienced Co-editor. While it is impossible to give absolute guarantees that such a situation will not happen again, we feel that the journal, its Editors, Co-editors and the Chester staff are now far better prepared to identify and challenge any further attempts to publish anything other than articles reporting genuine structural investigations in our journal. It is a strength of crystallography that fraudulent practices can be identified, even retrospectively, by diligent archiving of data and checking such as that carried out for the Union’s journals. We thank Ton Spek, George Ferguson and the IUCr Editorial Staff for all their input and assistance.

